# Ultrasound and computed tomography in the evaluation of mesenteric lesions: A pictorial review

**DOI:** 10.4102/sajr.v27i1.2595

**Published:** 2023-05-24

**Authors:** Snehal I. Kose, Sapna Singh, Anju Garg, Alpana Manchanda, Rajdeep Singh

**Affiliations:** 1 Department of Radiodiagnosis, Maulana Azad Medical College, Delhi, India; 2 Department of Surgery, Maulana Azad Medical College, Delhi, India

**Keywords:** CT, computed tomography, ultrasound, inflammatory myofibroblastic tumour, gastrointestinal stromal tumour

## Abstract

The mesentery is a broad fan-shaped fold of peritoneum that suspends the loops of small intestine from the posterior abdominal wall. Although primary neoplasms arising in the mesentery are rare, the mesentery is a major avenue for the dissemination of tumours, which can spread through hematogenous, lymphatic, direct or peritoneal seeding. Imaging helps in the diagnosis of these tumours and aids in directing appropriate treatment by assessing their size, extent and relationship with adjacent structures. The aim of this article is to describe the spectrum of imaging findings of the various mesenteric lesions using ultrasound and CT.

**Contribution:**

Evaluation of the mesentery is often neglected during routine ultrasound (US) because of inadequate training and unfamiliarity with the common US features encountered with mesenteric disease. CT plays an essential role in the diagnosis of mesenteric disease. Knowledge of imaging characteristics of various mesenteric lesions helps in timely diagnosis and management.

## Introduction

The mesentery is a broad, fan-shaped fold of peritoneum that suspends the loops of small intestine from the posterior abdominal wall. Secondary involvement of the mesentery from tumours elsewhere is much more common than primary mesenteric neoplasms such as desmoid tumour, inflammatory myofibroblastic tumour (IMFT), and others. Most patients with mesenteric lesions present with non-specific symptoms of abdominal pain, tenderness, palpable abdominal swelling, abdominal distension and weight loss. These symptoms are shared by pathologies of other abdominal organs and it is therefore very difficult to identify mesenteric lesions clinically.

Some mesenteric diseases present with distinctive imaging findings while others have similar findings, thereby complicating their differential diagnosis. Understanding the characteristic radiological patterns on ultrasound (USG) and CT offers valuable insights for differential diagnoses of mesenteric lesions and their treatment.

## Primary solid mesenteric lesions

Primary mesenteric solid neoplasms are rare and the majority of them are benign.

### Desmoid tumour

The most common soft tissue lesion in the mesentery is a desmoid tumour. It usually occurs in patients with familial adenomatous polyposis (FAP) or Gardner syndrome. Trauma or surgery serves as a predisposing factor for the development of desmoid tumours.^1^ On USG, this tumour appears as a well-defined or ill-defined mass with heterogenous echotexture and internal vascularity. On CT, it appears as a well-defined or ill-defined mass showing heterogenous enhancement usually in the portal venous phase and it may infiltrate the adjacent organs.^2^ The MRI appearance of desmoid tumours depends on the relative proportion of cellular, myxoid and fibrous components.

### Inflammatory myofibroblastic tumour

**FIGURE 1:**
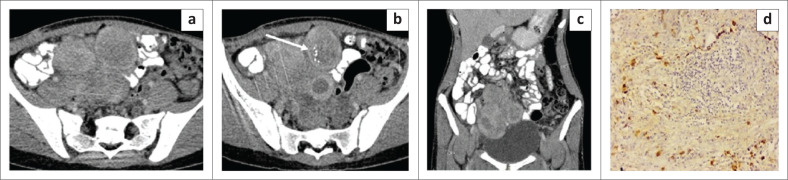
Mesenteric inflammatory myofibroblastic tumour. Axial (a, b) and coronal (c) contrast enhanced CT images of the abdomen in a 14-year-old girl reveal multiple well-circumscribed heterogenously enhancing nodular lesions in the mesentery indenting the dome of the urinary bladder. The lesions have a peripheral enhancing rim and central non-enhancing core. Few foci of punctate calcification (white arrow) are observed within one of the nodular lesions. Photomicrograph of the histopathological specimen on IgG4 immunochemistry (d) reveals spindle cells with areas of storiform fibrosis and infiltration by plasma cells (positive for IgG4).

Inflammatory myofibroblastic tumours usually occur in children and adolescents. They appear as solitary or multiple irregular hypoechoic masses with heterogeneous echotexture and increased echogenicity in the surrounding mesentery and omentum on USG and show increased internal vascularity on colour Doppler.^3^ Inflammatory myofibroblastic tumour appears as a hypo or iso-attenuating mass compared with skeletal muscle depending on the amount of myxoid or collagenous stroma, respectively. Dense central calcification may be noticed. They demonstrate various contrast enhancement patterns including early peripheral enhancement because of vascular tissue (Figure 1), delayed central enhancement of the fibrotic components, heterogenous, homogenous and absent enhancement.^4^ Differential diagnosis includes desmoid tumour and solitary fibrous tumour.

### Lipoma and liposarcoma

Lipomas are benign tumours composed of mature adipose tissue, usually associated with obesity and hyperlipidaemia. On USG, they appear as well-defined echogenic lesions without internal vascularity. On CT, they appear as a well encapsulated fatty attenuation mass. Differential diagnosis includes well-differentiated liposarcoma, which is characterised by larger size (usually greater than 10 cm), associated soft tissue component and thick irregular septations. Liposarcomas are rare malignant tumours of the mesentery. Liposarcomas are divided into five types histopathologically: well-differentiated, myxoid, pleomorphic, round cell and de-differentiated liposarcoma. Well-differentiated liposarcomas have mixed fatty and soft tissue components while de-differentiated liposarcomas have clearly separated soft tissue and fatty components. Myxoid liposarcomas have fluid density on CT with mixed soft tissue and fatty components. Pleomorphic and round cell liposarcomas have soft tissue components only, with no identifiable fatty component on imaging.

### Mesenteric Castleman disease

**FIGURE 2:**
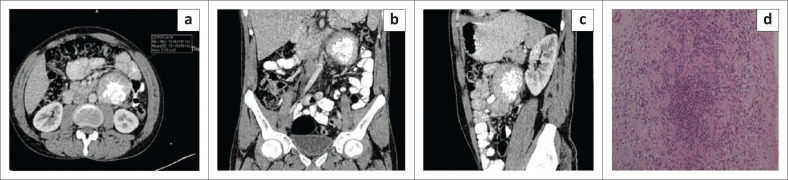
Mesenteric Castleman disease on histopathology. Axial (a), coronal (b) and sagittal (c) contrast enhanced CT images of the abdomen in a 26-year-old male reveal a well-defined round homogenously enhancing mass in the mesentery in the left lumbar region anterior to the lower pole of left kidney with a mean attenuation of ~101 HU and central arborising calcification. Photomicrograph of the histopathological specimen (d) shows acellular areas comprising vessels with dense sclerotic walls and cellular areas comprising spindle cells with bland nuclear morphology and inflammatory cell infiltrate comprising plasma cells.

Three patterns of mesenteric involvement have been described for Castleman disease, namely a solitary non-invasive mass (most common), a dominant infiltrative mass with associated lymphadenopathy and matted lymphadenopathy without a dominant mass. These tumours show early, homogenous and intense enhancement (Figure 2). Tumours measuring > 5 cm in size may show heterogenous enhancement. Calcification is seen in 10% of cases, which can be punctate or arborising in appearance. The arborising type of calcification (Figure 2) is characteristic of Castleman disease.^5^

### Calcifying fibrous tumour of the mesentery

**FIGURE 3:**
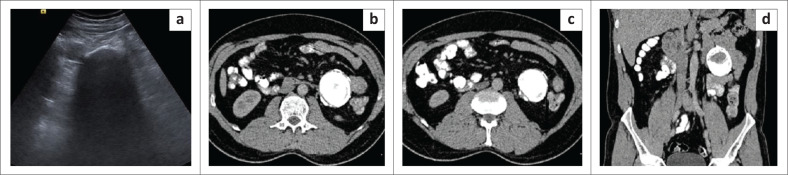
Calcifying fibrous tumour of the mesentery. Transverse grey scale ultrasound image (a) reveals a calcified lesion in left lumbar region causing extensive posterior acoustic shadowing. Axial contrast enhanced CT images (b, c) and coronal reformatted contrast enhanced CT image (d) reveal a well-defined oval smooth marginated mass lesion in the mesentery with near complete calcification, abutting the bowel loops.

Calcifying fibrous tumours appear as round hypo or hyperdense masses on CT with various patterns of calcification: irregular, scattered, punctate, band-like, clustered and amorphous^6^ (Figure 3).

### Malignant mesothelioma

**FIGURE 4:**
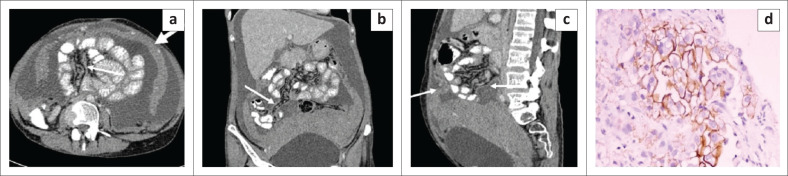
Malignant peritoneal mesothelioma. Contrast enhanced CT images – axial (a), coronal (b) and sagittal (c) reformatted CT images reveal gross ascites with a multilobulated heterogenously enhancing soft tissue mass lesion in the lower abdomen and pelvis. The lesion is encasing the uterus and bilateral adnexa and closely abutting the posterior and superior surface of urinary bladder. Sheet like thickening and enhancement of the parietal peritoneum, visceral peritoneum and leaves of mesentery is noted (thin white arrows) causing encasement of the bowel loops. Sheet like omental thickening (thick white arrows) and caking is seen. Mild stranding is observed in the mesenteric fat. Photomicrograph of the histopathological specimen shows cells arranged in a papillary pattern with a prominent fibrovascular core. On immunohistochemistry, these cells are positive for mesothelin (d).

Malignant mesotheliomas tend to spread as sheets of tissue over the parietal and visceral peritoneal surfaces and become confluent, encasing the abdominal organs (Figure 4). The ‘wet’ type presents as an irregular, nodular or sheet-like peritoneal thickening, omental mass and ascites while in the ‘dry’ type, peritoneal based masses are present with no ascites.^7^ Imaging presentation of this tumour is similar to peritoneal carcinomatosis. The presence of asbestos exposure, calcified pleural plaques and relatively less ascites compared with soft tissue burden, favours the diagnosis of malignant mesothelioma over peritoneal carcinomatosis.

### Primary mesenteric gastrointestinal stromal tumour

**FIGURE 5:**
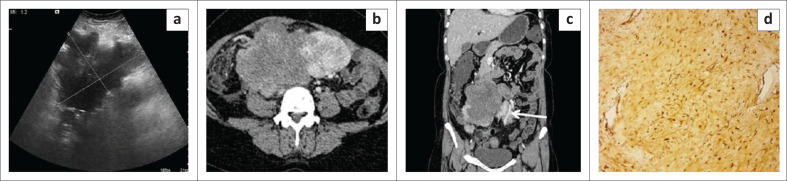
Primary mesenteric gastrointestinal stromal tumour. Transverse grey scale ultrasound image (a) in a 32-year-old female shows a large well-defined heterogenously hypoechoic mesenteric mass lesion measuring ~14 cm × 10 cm × 9.1 cm in size with lobulated margins. Axial (b) and coronal (c) contrast enhanced CT images reveal a large well-defined heterogenously enhancing mass lesion in the mesentery. The lesion shows lobulated margins and non-enhancing central necrotic area occupying > 50% of the area of tumour. No calcification, gas foci or cavitation is observed within the lesion. No direct invasion into surrounding bowel loops is noticed. An enhancing vessel is seen traversing through the mass (white arrow) Immunohistochemistry image of the surgical specimen (d) shows tumour cells positive for tumour marker for gastrointestinal stromal tumour (GIST), that is DOG-1 (discovered on GIST-1).

Extraintestinal gastrointestinal stromal tumours usually appear as well-defined masses with lobulated contours. They usually reveal heterogenous enhancement with central low attenuation areas occupying more than half of the tumour area (Figure 5) and increased peripheral attenuation. Central gas or extravasation of oral contrast may be seen due to communication of the mass with the bowel lumen.^8^ Calcification and lymphadenopathy are usually not present.

### Sclerosing mesenteritis

**FIGURE 6:**
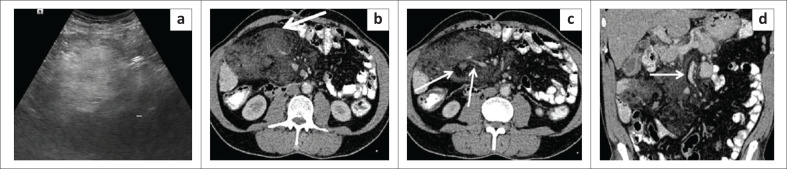
Mesenteric panniculitis. Transverse grey scale ultrasound image (a) of the abdomen in a 63-year-old male reveals diffusely echogenic mesentery with no well-defined solid mass. Axial contrast enhanced CT images of the abdomen (b, c) reveals an ill-defined inhomogenous mass of mean attenuation ~ 5.8 HU (greater than that of retroperitoneal fat). Multiple hyperdense nodules are seen within the mass and surrounding mesentery. Thin discontinuous slightly hyperattenuating soft tissue rim is seen on the anterior and posterior aspect of the mass limiting the mass from the surrounding mesentery (thick white arrow in b) – tumoural pseudocapsule sign. The mass is seen to encase superior mesenteric artery (SMA), superior mesenteric vein (SMV) and their branches with preservation of a ring of fat around mesenteric vessels and nodules (thin white arrows in c) – fat ring sign. Coronal reformatted CT image (d) reveals an ill-defined inhomogenous mass in the mesentery encasing the SMA, SMV and their branches with preservation of a rim of fat around SMA (thin white arrow – fat ring sign).

Mesenteric panniculitis presents as a well-defined fatty mass in the root of mesentery. Diffuse homogenous hyperechogenicity with good sound transmission through it and non-deviated vessels is seen on ultrasound. It appears as a well-defined mass of inhomogenous fatty tissue encasing the superior mesenteric vessels with no invasion of surrounding bowel loops on CT (Figure 6). Well-defined soft tissue nodules with preservation of fatty halo around vessels and nodules (fat ring sign) may be seen. Hyperattenuating stripe partly surrounding the mass (tumoural pseudocapsule sign) is seen in 59.2% cases.^9^

Retractile mesenteritis (sclerosing mesenteritis with predominant fibrotic component) is seen as one or more irregular fibrotic soft tissue mesenteric masses^7^, which may show calcification within and may encase adjacent bowel loops and vascular structures leading to signs of obstruction and hollow visceral ischaemia. An important differential for sclerosing mesenteritis is mesenteric metastases from carcinoid tumour, which also presents as a spiculated marginated mass in the mesentery; however, associated arterial phase hyperenhancing small bowel thickening is usually seen in carcinoid metastases.

## Primary cystic mesenteric lesions

### Lymphangiomas

**FIGURE 7:**
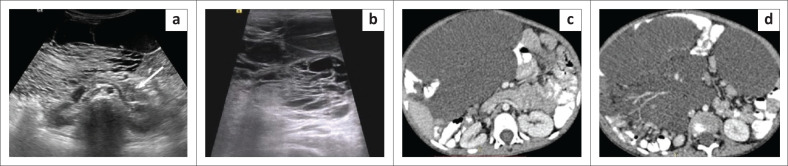
Mesenteric lymphangioma. Transverse grey scale ultrasound images (a, b) of the abdomen in a 3-year-old male child reveal an ill-defined multiloculated irregular-shaped thin-walled cystic lesion in the mesentery showing posterior acoustic enhancement. The lesion is located anterior to bilateral kidneys, pancreas (white arrow) and aorta. Multiple thin septations are noticed within the lesion creating a ‘cobweb’ appearance. Echogenic contents are seen within few loculi resulting in differential echogenicity of loculi. No calcification is noticed within the lesion. Axial contrast enhanced CT images (c, d) in the same patient reveal a multilobulated thin-walled fluid density cystic lesion in the mesentery anterior to pancreas and aorta. The lesion is insinuating between leaves of mesentery and bowel loops. Mesenteric vessels can be seen traversing through the lesion.

Lymphangiomas are benign lymphatic proliferations that usually occur in childhood. They appear as thin-walled cystic lesions with multiple thin septa (honeycomb or cobweb pattern) on USG and as a unilocular or multilocular cyst with enhancement of the wall or septations on contrast enhanced CT.^10^ They show variable attenuation depending on their contents (chylous, serous or haemorrhagic contents) and may insinuate between the mesenteric vessels (Figure 7).

### Simple mesothelial cysts

These appear as thin-walled anechoic unilocular cysts with posterior acoustic enhancement on USG and unilocular fluid attenuation lesions with no discernible wall on CT.^11^

### Benign cystic mesothelioma

**FIGURE 8:**
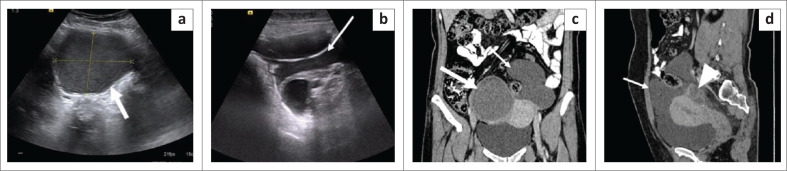
Benign cystic mesothelioma. Transverse grey scale ultrasound images (a, b) of the abdomen in a 38-year-old female reveal a well-defined cystic lesion around the right ovary with internal low-level echoes and a ground glass appearance suggestive of an endometrioma (white thick arrow in a). Another ill-defined thin-walled multiloculated cystic lesion (white thin arrow in b) is seen in midline and left side of lower abdomen and pelvis encasing the left ovary in its posterior aspect. Coronal CT image (c) of the abdomen reveals a well-defined hyperdense cyst of mean attenuation ~ 33 HU in the right adnexa (thick white arrow in c). Multilobulated fluid attenuation (~18 HU) cystic lesion with thin imperceptible walls is noticed in left lower abdomen and pelvis (thin white arrow). Sagittal reformatted CECT image (d) reveals a thin-walled multilobulated cystic lesion anterior and superior to uterus, encasing the left ovary posteriorly (arrowhead). Minimal fluid is observed within the endometrial cavity. No calcification or mural nodule is noted within the lesion.

Benign cystic mesotheliomas are also known as peritoneal inclusion cysts and they usually occur in premenopausal women. They are often associated with trauma, surgery, infection and endometriosis.^2^ They occur because of entrapment of fluid (secreted by the ovaries during ovulation) between peritoneal adhesions.^7^ They are seen as a fluid attenuation multilocular cystic lesion, multiple unilocular thin-walled cysts or unilocular cystic mass on CT with enhancing but non-calcified septa, which often surround the ovaries. On USG, they appear as unilocular or multilocular cystic lesions, which may be anechoic or may display contents of variable echogenicity (Figure 8).

### Benign mucinous cystic neoplasm of the mesentery

**FIGURE 9:**
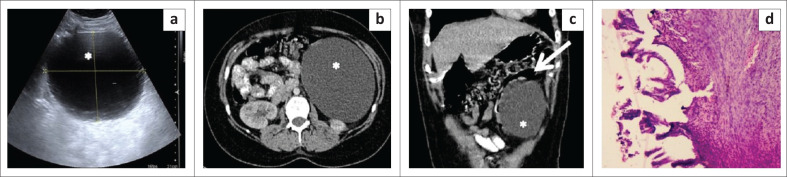
Mesenteric cyst. Grey scale ultrasound image (a) of the abdomen in a 26-year-old female reveal a well-defined thin walled unilocular anechoic cystic lesion (asterisks) showing posterior acoustic enhancement in the mesentery. No evidence of internal septations, calcification, mural nodule or internal vascularity noted. Axial contrast enhanced CT image of the abdomen (b) shows a well-defined thin walled unilocular cystic lesion (asterisks) anterior to lower pole of left kidney. Coronal reformatted contrast enhanced CT image (c) shows a well-defined unilocular cystic lesion (asterisks) in the left lumbar and iliac region displacing the descending colon medially (white arrow). Photomicrograph of the histopathological specimen (d) reveals the cyst wall lined by flattened and mucin secreting tall columnar epithelium. There is no atypia, mitosis or necrosis.

They usually arise from implantation of ovarian tissue during migration or metaplasia of mesothelial cells into ovarian tissue. They appear as thin-walled fluid attenuation unilocular cystic lesions in the mesentery (Figure 9).

## Secondary mesenteric lesions

Secondary involvement of the mesentery by tumours elsewhere is much more common than primary mesenteric tumours. These neoplasms spread to the mesentery by the following routes:

### Direct tumour spread

Several abdominal malignancies including biliary, pancreatic, gastric and colon cancers may invade directly into the mesentery.^12^

**FIGURE 10:**
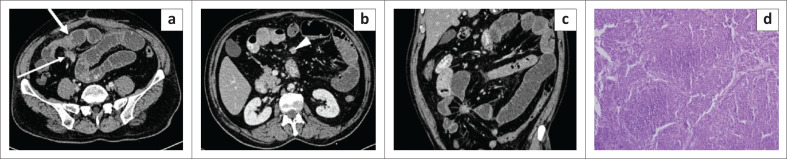
Carcinoid tumour of ileum with mesenteric metastases. Axial (a, b) and coronal (c) contrast enhanced CT images of the abdomen in a 56-year-old male reveal nodular enhancing wall thickening of the distal ileum (thick white arrow) causing obstruction and dilation of proximal small bowel loops. A homogenously enhancing soft tissue lesion (thin white arrow) is seen in the mesentery adjacent to the bowel thickening. Numerous fibrous strands are seen radiating from its periphery to the ileal loops with resultant kinking of ileal loops. An intensely enhancing nodular lesion is noted in the mesentery (arrowhead in b) likely a lymph node. Photomicrograph of the histopathological specimen from the mesenteric mass (d) reveals regular small polygonal cells in an insular pattern with round nuclei, salt and pepper chromatin and lacking atypia and mitosis.

Direct spread into the mesentery occurs in small bowel carcinoid, which appears as an intensely enhancing soft tissue mass with linear fibrous bands radiating in the surrounding fat. Calcification occurs in 70% cases.^13^ Associated thickening of the bowel wall and angulation of bowel loops might be noticed (Figure 10).

### Lymphatic spread

**FIGURE 11:**
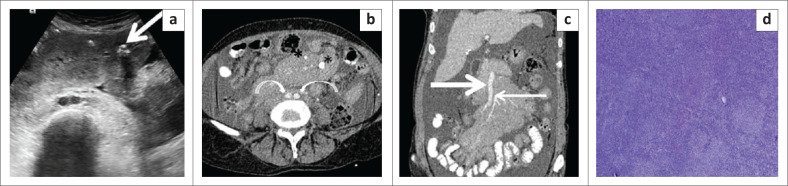
Non-Hodgkin’s lymphoma-follicular type. Transverse grey scale ultrasound image of the abdomen (a) in a 62-year-old female reveals a well-defined homogenously hypoechoic mass encasing the mesenteric vessels anterior to the common iliac arteries. A few foci of calcification are seen within the lesion (thick white arrow). Axial (b) and coronal (c) contrast enhanced CT images reveal a homogenously enhancing lymph nodal mass encasing the SMA (thin white arrow in c), superior mesenteric vein (thick white arrow in c) and their branches without causing luminal attenuation (sandwich sign). Another homogenously enhancing lymph nodal mass lesion is seen encasing the common iliac arteries (curved arrows in b). A few foci of calcification are seen within the lesion (asterisk in b). Gross ascites is also seen. Photomicrograph of the histopathological specimen (d) shows variable sized follicles replacing the entire lymph node. The follicles are comprised of follicular centre cells with an admixed large number of centroblasts (H&E, 200x).

Lymphoma appears as a lobulated sonolucent mass surrounding vascular trunks with maintained perivascular echogenic fat giving the ‘sandwich appearance’.^14^ Lymph nodes may be small and discrete in early disease and later coalesce to form a conglomerate soft tissue mass, which grows around and displaces surrounding structures. Involved lymph nodes have soft tissue attenuation (40 HU – 50 HU) and demonstrate homogeneous enhancement following contrast administration (Figure 11). Besides lymphoma, many other tumours such as lung, breast, colon, ovarian cancers, chronic lymphoid leukaemia, and others, can involve the mesentery via the lymphatics.

### Hematogenous spread

Melanoma, breast and lung carcinoma can involve the mesentery via the hematogenous route.^12^ These lesions are usually seen along the antimesenteric border because of the abundant submucosal vascular plexus.

Four patterns of peritoneal seeding are noted:^12^

**FIGURE 12:**
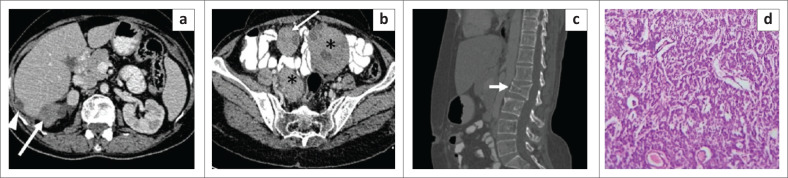
Bilateral ovarian granulosa cell tumour with metastatic deposits. Axial contrast enhanced CT image (a) of the abdomen in a 45-year-old female reveals multiple heterogeneously enhancing predominantly solid nodular deposits in Morison’s pouch (thick white arrow) and along the lateral surface of the liver (arrowhead). Axial section in the pelvis (b) reveals heterogeneously enhancing, predominantly solid masses in both ovaries (asterisk) along with a nodular deposit in the mesentery (thin white arrow). Sagittal reformatted CT image on bone window (c) shows a fracture and collapse of the L2 vertebral body (short thick white arrow), likely a pathological fracture related to metastases. Photomicrograph of the histopathological specimen (d) of the mesenteric lesion [H & E stain] reveals scattered and clustered round plasmacytoid cells with central to eccentrically placed nuclei and a prominent microfollicular pattern. Some of the follicles show central pinkish material (Call–Exner bodies) and moderate focal pleomorphism.

- *Infiltrative pattern* leading to a misty mesentery.
- *Nodular pattern/caking* (Figure 12).
- *Retractile pattern* seen as small bowel retraction, angulation and kinking.
- *Stellate mesentery*: The straightened mesenteric blood vessels are held rigid within a thickened sheet such as the mesentery, producing the characteristic stellate appearance on CT because of infiltration of tumour along the mesenteric vessels.

## Infections

### Abdominal tuberculosis

Abdominal lymphadenopathy is the most common finding associated with tuberculosis. Involved lymph nodes appear enlarged, conglomerate and show ring enhancement (necrosis). Other findings observed in cases of abdominal tuberculosis include mesenteric nodularity, fat stranding, peritoneal thickening, localised collections, free fluid, clumping of bowel loops, bowel wall thickening and solid organ involvement in the form of hepatomegaly, splenomegaly, liver and splenic granulomas.^15^

### Whipple’s disease

It is an infectious condition caused by Gram-positive bacterium, *Tropheryma whipplei*. On imaging, small bowel wall thickening associated with enlarged mesenteric lymph nodes demonstrating hypoattenuating centres because of fat deposition can be seen. Diagnosis is made by small intestinal mucosal biopsy.^16^

### Actinomycosis

*Actinomyces israelii* is an anaerobic Gram-positive bacterium and is a normal inhabitant of the oral cavity, gastrointestinal tract and female genital tract. It spreads to involve the mesentery when a mucosal breach is present such as inflammation, surgery, trauma or intrauterine contraceptive device (IUCD) use.^17^ On imaging, an ill-defined heterogeneously enhancing soft tissue mass infiltrating the mesentery and adjacent organs can be seen. Septic embolism leading to hepatic, renal and splenic abscesses can occur.^2^ Diagnosis is made based on aggressive imaging features and subtle clinical symptoms in predisposed individuals. Biopsy should be performed in indeterminate imaging findings, which reveal sulphur granules representing bacterial colonies.

### Hydatid cyst

**FIGURE 13:**
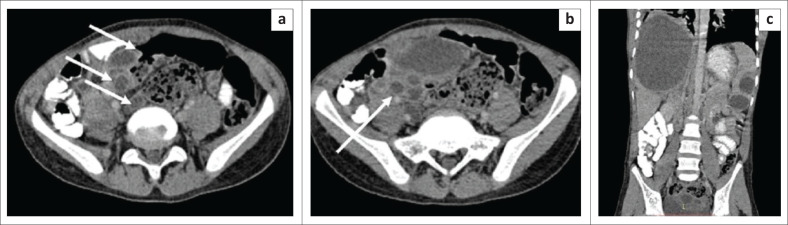
Hydatid cysts. Axial contrast enhanced CT images (a, b) of the abdomen in a 6-year-old child reveal multiple, well-defined peripherally enhancing unilocular cystic lesions in the mesentery and pelvis (white arrows) with no internal septations, mural nodule or calcification within the lesions. Coronal reformatted CT image (c) reveals well-defined unilocular cysts in the liver and spleen with no internal septations, calcifications or mural nodule.

These usually occur because of intraperitoneal rupture of liver or splenic hydatid cysts (Figure 13). Hydatid cysts can appear as either unilocular cysts (Type I cyst), multiloculated cystic lesions with daughter cysts at the periphery (type IIA), multilocular lesions with irregular daughter cysts occupying almost the entire volume of the maternal cyst (rosette appearance – type IIB) or high attenuation lesion containing occasional calcification and daughter cysts (type IIC). Type III cysts are calcified cysts while complicated hydatid cysts are included in the type IV category.^18^

## Inflammatory bowel disease

**FIGURE 14:**
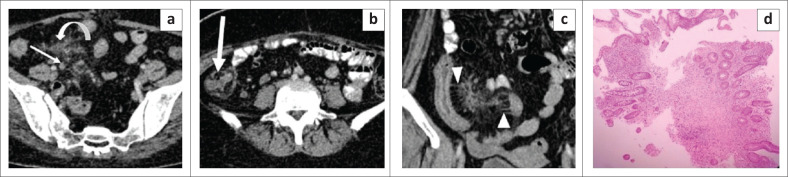
Crohn’s disease. Axial (a, b) and coronal (c) contrast enhanced CT images of the abdomen in a 45-year-old female reveal mesenteric fibrofatty proliferation in the right iliac fossa (curved white arrow), mesenteric lymph nodes (thin white arrow), thickening of the caecal wall with submucosal fat proliferation ([fat target sign] – thick white arrow) and prominent and straightened vasa recta ([Comb sign] – arrowheads). Photomicrograph of the ileal biopsy specimen (d) shows transmural infiltration of the ileum by inflammatory cells with loss of the villous architecture.

Mesenteric changes in inflammatory bowel disease include mesenteric fibrofatty proliferation, mesenteric lymphadenopathy, fat stranding (Figure 14), abscess and prominent vasa recta (Comb sign).

### Vascular anomalies

#### Acute mesenteric ischaemia

**FIGURE 15:**
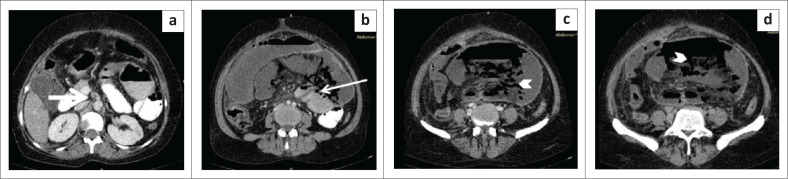
Superior mesenteric artery (SMA) thrombosis with acute mesenteric ischaemia. Axial contrast enhanced CT images (a–d) reveal thrombus in the superior mesenteric artery (thick white arrow in a) at the level of its origin from aorta. The large and small bowel loops appear dilated and show paper-thin walls. Few air foci are seen within wall bowel loops suggestive of pneumatosis intestinalis (thin white arrow in b). Air is also noticed along the mesenteric vessels (arrowheads in c and d).

Findings in acute mesenteric ischaemia related to superior mesenteric artery (SMA) thrombosis include dilated bowel loops with paper-thin walls, pneumatosis intestinalis, air within mesenteric vessels, mesenteric fat stranding and free fluid on CECT^19^ (Figure 15).

#### SMA-SMV arteriovenous fistula

On USG, tangled serpentine vessels can be seen, which show pulsatile waveforms on spectral doppler while on CECT, tangled serpentine vessels can be seen with early opacification of venous channels in the arterial phase. Direct communication between branches of the SMA and superior mesenteric vein (SMV) can be visualised on volume rendering technique (VRT) images.

## Role of imaging

Ultrasound is the primary imaging modality in the evaluation of mesenteric lesions. It helps in characterising mesenteric lesions as solid or cystic. Ultrasound guided percutaneous biopsy offers real time assessment of the needle tract to avoid vascular injury while performing biopsy.^20^ Evaluation of lesions is limited on USG because of operator-dependence, obscuration of the mass in the presence of a gaseous abdomen and obese patients, and inadequate visualisation of the extent of large mesenteric lesions. Contrast enhanced CT is the primary workhorse for evaluation of mesenteric lesions. It helps in determining the origin of the lesions, providing thoughtful differential diagnosis of masses, for selecting the site of biopsy, determining the extent of pathology and accessibility for resectability of lesion. CT, however, has several limitations including poor soft tissue contrast resolution, exposure to ionising radiation and contrast reactions. MRI can be used as the next step in characterising mesenteric lesions. It has higher soft tissue contrast resolution and helps in characterising fluid content of cysts as serous, mucinous, chylous or haemorrhagic and the soft tissue component of solid lesions. Fluoro-deoxyglucose (FDG) positron emission tomography (PET) CT is presently used to detect lymph nodes and distant metastases in mesenteric tumours and also for response assessment to chemotherapy.

## Management of mesenteric lesions

Management of mesenteric lesions depends on symptoms and imaging findings. Mesenteric lesions detected incidentally on imaging need classification as benign or malignant. Definite diagnosis of a few pathologies such as mesenteric panniculitis, lymphoma, desmoid tumour in patients with FAP and benign cystic lesions can be made on imaging. However, lesions with indeterminate imaging findings need to be biopsied to rule out malignancy. Imaging helps in determining the site for percutaneous biopsy in such tumours. Lesions that are inaccessible to percutaneous biopsy or show indeterminate histopathology findings on percutaneous biopsy require surgical biopsy to confirm the diagnosis.^21^

Once confirmed by biopsy, management of the mesenteric lesions depends on the histopathology. Lymphomas and desmoid tumours are managed conservatively with chemotherapy and hormonal therapy or imatinib, respectively. Well-circumscribed lesions located in the periphery of the mesentery can be resected completely, without sacrificing significant bowel length or major mesenteric vessels. Infiltrative mesenteric lesions, located in the root of mesentery are usually managed conservatively because of involvement of major vessels and the need for sacrificing a major portion of bowel, which may lead to small bowel syndrome. Well-defined malignant tumours and symptomatic benign tumours are treated surgically. The goal of surgery is R0 resection (grossly as well as microscopically negative tumour margins), while R1 resection followed by adjuvant chemoradiotherapy can also be performed. Well-defined asymptomatic benign lesions can be followed up and resection is performed when they become large or cause symptoms. Symptomatic infiltrative benign or malignant lesions can be managed by debulking surgery to reduce tumour load and prevent complications such as bowel obstruction or mesenteric ischemia.

## Conclusion

Although histopathology is the gold standard for diagnosis of mesenteric lesions, imaging plays an important role in diagnosis of mesenteric neoplasms, detection of complications and in deciding appropriate treatment options. Ultrasound is inadequate for optimal visualisation of mesenteric neoplasms and in detecting their relation with other structures because of shadowing from bowel gas, calcification or inadequate penetration of sound beams in obese patients. CT is an excellent imaging modality in the characterisation of mesenteric lesions, detecting their extent and relations with surrounding structures, which is useful for surgical planning. However, CT too has several limitations and other imaging modalities such as MRI and FDG-PET can be helpful in further characterisation of mesenteric lesions.

## References

[1] Shields CJ, Winter DC, Kirwan WO, Redmond HP. Desmoid tumours. Eur J Surg Oncol. 2001;27(8):701–706. 10.1053/ejso.2001.1169 11735163

[2] Dufay C, Abdelli A, Le Pennec V, Chiche L. Mesenteric tumors: Diagnosis and treatment. J Visc Surg. 2012;149(4):e239–e251. 10.1016/j.jviscsurg.2012.05.005 22796300

[3] Qian J, Zhu K, Ye J. Ultrasonic manifestations of mesenteric inflammatory myofibroblastic tumors in children. Front Pediatr. 2019;7:39. 10.3389/fped.2019.00039 30891434 PMC6411639

[4] Chung EM, Biko DM, Arzamendi AM, Meldrum JT, Stocker JT. Solid tumors of the peritoneum, omentum, and mesentery in children: Radiologic-pathologic correlation. Radiographics. 2015;35(2):521–546. 10.1148/rg.352140273 25763737

[5] Bonekamp D, Horton KM, Hruban RH, Fishman EK. Castleman disease: The great mimic. Radiographics. 2011;31(6):1793–1807. 10.1148/rg.316115502 21997995

[6] Chorti A, Papavramidis TS, Michalopoulos A. Calcifying fibrous tumor: Review of 157 patients reported in international literature. Medicine. 2016;95(20):e3690. 10.1097/MD.0000000000003690 27196478 PMC4902420

[7] McLaughlin PD, Filippone A, Maher MM. Neoplastic diseases of the peritoneum and mesentery. Am J Roentgenol. 2013;200(5):W420–W430. 10.2214/AJR.12.8494 23617509

[8] Kim HC, Lee JM, Kim SH, et al. Primary gastrointestinal stromal tumors in the omentum and mesentery: CT findings and pathologic correlations. Am J Roentgenol. 2004;182(6):1463–1467. 10.2214/ajr.182.6.1821463 15149991

[9] Daskalogiannaki M, Voloudaki A, Prassopoulos P, et al. CT Evaluation of mesenteric panniculitis: Prevalence and associated diseases. Am J Roentgenol. 2000;174(2):427–431. 10.2214/ajr.174.2.1740427 10658720

[10] Gunasekaran G, Naik D, Kandhasamy SC, Soren DN. Giant mesenteric cystic lymphangioma: A rare cause of intra-abdominal catastrophe. Int Surg J. 2019;6(11):4184–4186. 10.18203/2349-2902.isj20195152

[11] Ros PR, Olmsted WW, Moser RP, Dachman AH, Hjermstad BH, Sobin LH. Mesenteric and omental cysts: Histologic classification with imaging correlation. Radiology. 1987;164(2):327–332. 10.1148/radiology.164.2.3299483 3299483

[12] Nougaret S, Lakhman Y, Reinhold C, et al. The wheel of the mesentery: Imaging spectrum of primary and secondary mesenteric neoplasms – How can radiologists help plan treatment?: Resident and fellow education feature. RadioGraphics. 2016;36(2):412–413. 10.1148/rg.2016150186 26963454 PMC4901984

[13] Sheth S, Horton KM, Garland MR, Fishman EK. Mesenteric neoplasms: CT appearances of primary and secondary tumors and differential diagnosis. Radiographics. 2003;23(2):457–473. 10.1148/rg.232025081 12640160

[14] Mueller PR, Ferrucci Jr, JT, Harbin WP, Kirkpatrick RH, Simeone JF, Wittenberg J. Appearance of lymphomatous involvement of the mesentery by ultrasonography and body computed tomography: The ‘sandwich sign’. Radiology. 1980;134(2):467–473. 10.1148/radiology.134.2.7352232 7352232

[15] Burrill J, Williams CJ, Bain G, Conder G, Hine AL, Misra RR. Tuberculosis: A radiologic review. Radiographics. 2007;27(5):1255–1273. 10.1148/rg.275065176 17848689

[16] Misbah SA, Aslam A, Costello C. Whipple’s disease. Lancet. 2004;363(9409):654–656. 10.1016/S0140-6736(04)15600-6 14987893

[17] Pusiol T, Morichetti D, Pedrazzani C, Ricci F. Abdominal- pelvic actinomycosis mimicking malignant neoplasm. Infect Dis Obstet Gynecol. 2011;2011:747059. 10.1155/2011/747059 21904441 PMC3163399

[18] Mehta P, Prakash M, Khandelwal N. Radiological manifestations of hydatid disease and its complications. Trop Parasitol. 2016;6(2):103–112. 10.4103/2229-5070.190812 27722098 PMC5048696

[19] Florim S, Almeida A, Rocha D, Portugal P. Acute mesenteric ischaemia: A pictorial review. Insights Imaging. 2018;9:673–682. 10.1007/s13244-018-0641-2 30120722 PMC6206376

[20] Lee JK, Baek SY, Lim SM, Lee KH. Reticular infiltrations alone without mass in the mesentery and omentum identified at contrast-enhanced CT: Efficacy of US-guided percutaneous core biopsy. Radiology. 2011;261(1):311–317. 10.1148/radiol.11103523 21873256

[21] Gottlieb RH, Tan R, Widjaja J, et al. Extravisceral masses in the peritoneal cavity: Sonographically guided biopsies in 52 patients. Am J Roentgenol. 1998;171(3):697–701. 10.2214/ajr.171.3.9725299 9725299

